# Up-Regulation of the Biosynthesis and Release of Substance P through Wnt/β-Catenin Signaling Pathway in Rat Dorsal Root Ganglion Cells

**DOI:** 10.1371/journal.pone.0129701

**Published:** 2015-06-08

**Authors:** Yu-Sang Li, Yang Xi, Xiao-Jun Li, Chang-Long Leng, Mei-Mei Jia, Wei Kevin Zhang, He-Bin Tang

**Affiliations:** 1 Department of Pharmacology, College of Pharmacy, South-Central University for Nationalities, No. 182 Minyuan Road, Hongshan-qu, Wuhan 430074, China; 2 Medical School, Ningbo University, No. 818 Fenghua Road, Jiangbei District, Ningbo 315211, China; University of Texas Medical Branch, UNITED STATES

## Abstract

To examine regulatory effects of β-catenin on the biosynthesis and release of substance P, a rat chronic constriction injury (CCI) model and a rat dorsal root ganglion (DRG) cell culture model were used in the present study. The CCI treatment significantly induced the overall expression of β-catenin (158 ± 6% of sham) in the ipsilateral L5 DRGs in comparison with the sham group (109 ± 4% of sham). The CCI-induced aberrant expression of β-catenin was significantly attenuated by oral administration of diclofenac (119 ± 6% of the sham value; 10 mg/kg). Importantly, aberrant nuclear accumulation of β-catenin in cultured DRG cells resulted in up-regulation of the *PPT-A* mRNA expression and the substance P release. The up-regulation of both the *PPT-A* mRNA expression and the substance P release by either a GSK-3β inhibitor TWS119 (10 μM) or a Wnt signaling agonist Wnt-3a (100 ng/ml) were significantly abolished by an inhibitor of cyclooxygenase-2 (COX-2; NS-398, 1 μM). Collectively, these data suggest that nociceptive input-activated β-catenin signaling plays an important role in regulating the biosynthesis and release of substance P, which may contribute to the inflammation responses related to chronic pain.

## Introduction

Substance P, encoded by the *preprotachykinin-A* (*PPT-A*) gene, is synthesized in the dorsal root ganglion (DRG) neurons. The nociceptive stimuli-evoked release of substance P from cultured DRG cells could be significantly attenuated by the inhibition of COX-2 [1–3]. Intrathecal injection of substance P induced spinal prostaglandin E_2_ release and thermal hyperalgesia can also be reversed by spinal COX-2 inhibition [4,5]. Moreover, the deletion of the *PPT-A* gene could reduce the stimulus-induced surface insertion of delta-opioid receptors and abolish delta-opioid receptor-mediated spinal analgesia and morphine tolerance [6]. Hence, substance P is an important neurotransmitter released from primary afferent neurons for conveying nociceptive information and transmitting pain [7].

As a key mediator in the Wnt signaling pathway, β-catenin regulates multiple cellular functions, including proliferation, differentiation, inflammation and oncogenesis [8–10]. Recent studies indicated that nerve injury caused a rapid-onset and long-lasting expression of Wnt-3a, as well as activation of Wnt/β-catenin signaling in the primary sensory neurons, and induced an early up-regulation of β-catenin protein expression in the dorsal horn of the rat spinal cord [11–13]. Moreover, prostaglandin E_2_, a cyclooxygenase (COX) product contributing to inflammatory pain, has been reported to induce the accumulation of nuclear β-catenin through inactivating glycogen synthase kinase 3β (GSK-3β) [14,15]. Based on the above observations, we anticipated that the cytoplasmic/nuclear β-catenin accumulation might be an important regulator of nociceptive signaling in the process of chronic pain.

The above-mentioned observations strongly support that the β-catenin signaling might serve as an important regulator in the process of chronic pain. To uncover the novel molecular mechanisms underlying chronic pain, we investigated the effect of β-catenin nuclear accumulation on the biosynthesis and release of substance P in the DRG cells of chronic constriction injury (CCI) rats. A further examination of their relationship was demonstrated in cultured DRG cells by up- or down-regulating the Wnt/β-catenin signaling by various activators (GSK-3β inhibitor TWS119 or Wnt signaling agonist Wnt-3a) or inhibitors (NS-398, a COX-2 inhibitor; Dkk1, an antagonist of the Wnt/β-catenin signaling), respectively.

## Materials and Methods

### Animal care

The care and use of animals and experimental protocols for this study were performed according to the Guide for Animal Experimentation, South-Central University for Nationalities and the Committee of Research Facilities for Laboratory Animal Sciences, South-Central University for Nationalities, China. The protocols were approved by the Committee on the Ethics of Animal Experiments of the South-Central University for Nationalities, China (Permit Number: 2011-SCUEC-AEC-001). All surgery was performed under trichloroacetaldehyde monohydrate (450 mg/kg, i.p) anesthesia, and the Wistar rats for DRG neurons were sacrificed by decapitation after being anesthetized by CO2. All efforts were made to minimize suffering.

### Peripheral nerve injury

The chronic constriction injury of the sciatic nerve had been performed in adult male Wistar rats as in our previous study [16]. To investigate effects of β-catenin on the nerve injury-induced pain responses, we therefore used the same paraffin-embedded L5 DRG specimens of CCI rats described in our previous study [16] and the isolated L5 DRGs from the sham-operated and CCI rats treated with or without diclofenac in an additional experiment. To isolate the L5 DRGs, ten rats in each group were sacrificed via rapid decapitation four weeks after the operation. The 30 samples of isolated L5 DRGs in an additional experiment were equally divided and used for RT-PCR and Western blot analysis.

### Immunofluorescence staining

Immunofluorescence analysis of β-catenin localization was performed with anti-β-catenin polyclonal antibody (1:100 dilution; Cayman Chemical, Ann Arbor, MI) on 4-μm paraffin-embedded L5 DRG sections and paraformaldehyde-fixed cultured DRG cell sections [16]. Then, the sections were incubated overnight at 4°C followed by incubation for 60 min at room temperature with Alexa Fluor 488 goat anti-rabbit IgG (1:1,000; Molecular Probes, Eugene, OR, USA). Finally, the immunofluorescence quantification for β-catenin was performed by an inverted fluorescence microscope (Eclipse Ti, Nikon, Japan) with the use of image analysis software (NIS-Elements AR 3.0, Nikon) from 4 rats in each group according to previously described method [16].

### Cell treatment and measurement of the substance P content

The isolated DRG cells from adult Wistar rats (6–9 weeks of age) were cultured at 37°C in a water-saturated atmosphere with 5% CO_2_ for 5 days before the initiation of experiments [3]. Next, the DRG cells (5–6 DRGs/35 mm dish) were separately exposed to Dkk1 (R&D Systems, Minneapolis, MN 55413, USA) or NS-398 (Sigma Chemical Co., St Louis, MO, USA) in serum-free DMEM containing peptidase inhibitors (1 μM phosphoramidon, 4 μg/ml bacitracin and 1 μM captopril; Sigma) for 15 min at 37°C in a water-saturated atmosphere with 5% CO_2_. Thereafter, the cells pretreated with various drugs were continuously stimulated with TWS119 (Cayman Chemical, Ann Arbor, MI) or Wnt-3a (R&D Systems) for 1, 6, 24 or 48 h in DMEM at 37°C. The DRG cells treated by peptidase inhibitors alone were used as a control. The substance P content in the cultured rat DRG cells was measured using a highly sensitive radioimmunoassay as previously described [13].

### Real-time PCR

Total RNA harvested from the isolated L5 DRGs of rats treated with or without CCI of the sciatic nerve and the cultured DRG cells treated with or without TWS119 or Wnt-3a according to the manufacturer’s instructions (The RNeasy Micro kit; Qiagen GmbH, Hilden, Germany) and an acid guanidinium thiocyanate-phenol-chloroform extraction method [17] was separately subjected to reverse transcription into cDNA by using a Superscript kit (TaKaRa Bio, Dalian, China) according to the manufacturer’s protocol. Quantitative real-time PCR was performed on a Thermal Cycler Dice TP800 system (TaKaRa Bio, Japan) using SYBR Premix Ex Taq II (Takara Bio, Dalian, China) with 40 cycles of 95°C for 5 s and 60°C for 30s. *GAPDH* was used as an internal standard. The following primer pairs were used: β-catenin, 5’-tggacaatggctactcaagctgac-3’ and 5’-gggatgagcagcgtcaaactgcgt-3’; COX-2, 5’-tgtatgctaccatctggcttcgg-3’ and 5’-gtttggaacagtcgctcgtcatc-3’; *PPT-A*, 5’-ggtgccaacgatgatct-3’ and 5’-gcatcccgtttgcccatt-3’; *GAPDH*, 5’-tgtgtccgtcgtggatctga-3’ and 5’-ttgctgttgaagtcgcaggag-3’.

### Western blot analysis

The cell membranous, cytoplasmic and nuclear proteins were extracted from cultured DRG cells using a Nucl-Cyto-Mem Preparation Kit, according to the manufacturer’s instructions (Applygen, Beijing, China). The total proteins of the isolated L5 DRGs were extracted according to the manufacturer’s instructions (a Western & IP Cell Lysis Kit; Beyotime, Haimen, China). The protein concentrations of each fraction were determined using a Lowry protein assay [1]. Then equal amounts of protein were loaded onto 10% sodium dodecyl sulfate polyacrylamide gel electrophoresis and transferred to a polyvinylidene fluoride membrane. The membranes were probed with the following primary antibodies: anti-β-catenin polyclonal antibody (1:1000 dilution; Cayman) and the mouse monoclonal antibody for β-actin (1:5000 dilution; Sigma). The horseradish peroxidase-conjugated anti-rabbit and anti-mouse secondary antibodies (1:2000 dilution; Cell Signaling Technology, Beverly, MA) were used for chemiluminescence detection according to the manufacturer’s instructions, respectively.

### Statistical analysis

Results are shown as the means ± SEM. The statistical analyses were performed by one- or two-way ANOVA as indicated in the text using Instat software (GraphPad Prism 5, USA). A *P* value less than 0.05 was considered statistically significant.

## Results

### Nuclear accumulation of β-catenin in DRG cells of chronic constriction injury rats

**Fig 1:**
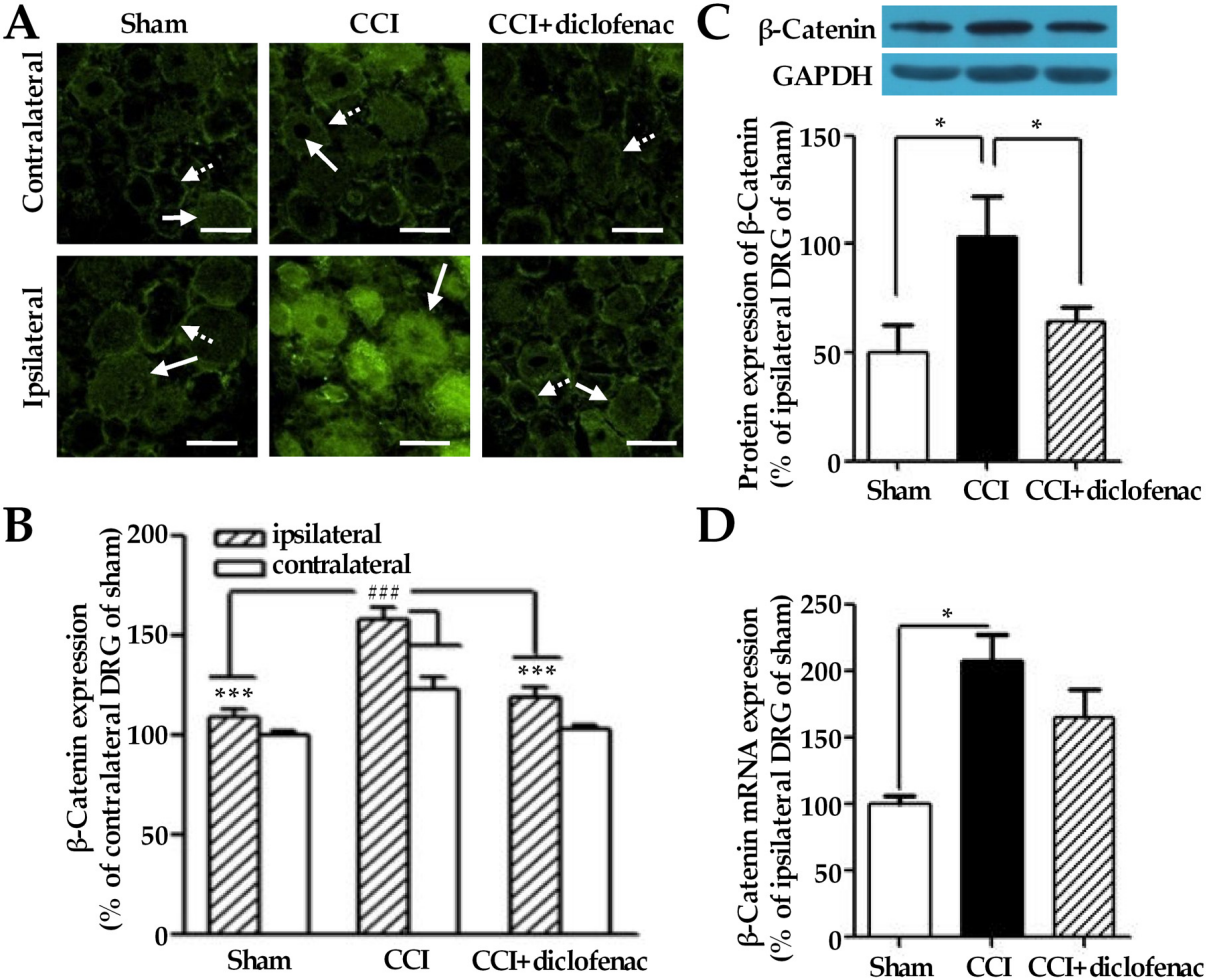
Changes of β-catenin protein expression and its gene transcription level in the L5 DRGs of CCI rats. (A) Representative immunofluorescence photomicrographs of β-catenin (Green) in the ipsilateral L5 DRGs of CCI rats treated with or without diclofenac (10 mg/kg) on the 28^th^ day after the operation. Dashed arrows denote cells of membrane only localized β-catenin; solid arrows denote cells of mixed localization of β-catenin. (B) Fluorescence determination of the β-catenin expression in the L5 DRGs of (A). The L5 DRG specimens were obtained from the same paraffin-embedded L5 DRG sections of CCI rats shown in our previous study [16]. (C) Representative blots and summary of the β-catenin expression in the ipsilateral L5 DRGs of CCI rats treated with or without diclofenac. (D) The gene transcription level of *β-catenin* in the ipsilateral L5 DRGs of CCI rats treated with or without diclofenac. The isolated L5 DRGs from the sham-operated and CCI rats treated with or without diclofenac in an additional experiment. ## denotes *P* < 0.01 versus the contralateral L5 DRG of rats with CCI alone; *, *** denote *P* < 0.05, 0.001 versus the ipsilateral L5 DRG of rats with CCI alone (n = 4 for the data of fluorescence determination; n = 5 for the data of RT-PCR and Western blot analysis; one-way analysis of variance, followed by Newman-Keuls post hoc test). Scale bars: 25 μm.

We had previously demonstrated that the CCI treatment induced hyperalgesia in the ipsilateral hind paw accompanying with up-regulation of both COX-2 protein expression and the biosynthesis and release of substance P in the ipsilateral L5 DRGs of rats [16]. Herein, we sought to investigate β-catenin expression and its nuclear accumulation in L5 DRG cells of CCI rats using the remaining sections of the same paraffin-embedded L5 DRG specimens from CCI rats in our previous study [16]. As shown in Fig 1, CCI treatment significantly induced total expression of β-catenin (158 ± 6% of sham, n = 4) in ipsilateral L5 DRGs in comparison to ipsilateral L5 DRGs of sham group (109 ± 3% of sham, n = 4) on the 28^th^ day after the operation. The total level of β-catenin expression (123 ± 6% of sham, n = 4) in contralateral L5 DRGs of CCI group was also higher than that in contralateral L5 DRGs of sham (100 ± 3% of sham, n = 4). Moreover, the CCI-induced total expression of β-catenin in ipsilateral L5 DRGs was significantly inhibited by diclofenac (109 ± 3% of sham, n = 4) in ipsilateral L5 DRGs (Fig 1B).

On the other hand, the percentage of cells that had membrane-only localization (indicated as M) and whole cell localization (indicated as M+C+N) of β-catenin in the same specimens of DRGs described above were calculated according to a randomly selected field in each image (Table 1). The nuclear accumulation of β-catenin was accompanied with its increased expression in cytosol of L5 DRG cells from CCI rats (Fig 1A and 1B, Table 1). Therefore, the localization of β-catenin expressed in cytoplasmic/nuclear of DRG cells was herein considered as the nuclear accumulation of β-catenin. As shown in Table 1, CCI treatment significantly induced β-catenin nuclear accumulation (88 ± 9% of total number of β-catenin-positive cells, n = 4) in comparison to that (57 ± 4% of total number of β-catenin-positive cells, n = 4) of sham in ipsilateral L5 DRGs. Moreover, the CCI-induced nuclear accumulation of β-catenin was significantly inhibited by diclofenac (59 ± 7% of total number of β-catenin-positive cells, n = 4) in ipsilateral L5 DRGs (Table 1).

**Table 1:** The percentage of the cells expresing β-catenin in the membrane, cytoplasm and nuclei of the L5 DRGs shown in Fig 1A.

Beta-catenin expressions				
	Ipsilateral DRGs		Contralateral DRGs	
	M	M+C+N	M	M+C+N
Sham	43.0 ± 3.6	57.0 ± 3.6	40.0 ± 6.2	60.0 ± 6.2
CCI	87.5 ± 8.5^a^	87.5 ± 8.5^a^	65.1 ± 3.4^a^	34.9 ± 3.4^a^
CCI+diclofenac	40.9 ± 6.8^b^	59.1 ± 6.8^b^	72.3 ± 9.2	27.7 ± 9.2

The L5 DRG specimens were obtained from the same paraffin-embedded L5 DRG sections of CCI rats shown in our previous study [16]. The data were obtained from four rats in each group.

^a^ denotes *P* < 0.01 versus the contralateral L5 DRGs of sham rats;

^b^ denotes *P* < 0.01 versus the ipsilateral L5 DRGs of CCI rats.

Here, to further know whether the CCI treatment surely elicits the overall expression of β-catenin in the ipsilateral L5 DRGs of rats, we have measured its mRNA and protein expression levels by RT-PCR and Western blot analysis in an additional experiment. As expected, the CCI treatment certainly elicited the overall expression of β-catenin (206 ± 37% of sham for protein; 207 ± 21% of sham for mRNA; n = 5) on the 28^th^ day after the operation (Fig 1C). Consistent with the findings by immunofluorescence staining, the CCI-elicited mRNA and protein expression levels of β-catenin were partially inhibited by diclofenac (128 ± 14% of sham for protein; 166 ± 21% of sham for mRNA; n = 5) in ipsilateral L5 DRGs (Fig 1D). All together, the results shown in Fig 1 and Table 1 indicate that damage to peripheral nerve induces β-catenin nuclear accumulation and its overall expression in ipsilateral L5 DRGs in a diclofenac-sensitive manner.

### Reciprocal regulation between β-catenin and COX-2 in DRG cells after exposure to activators of Wnt/β-catenin signaling pathway

**Fig 2:**
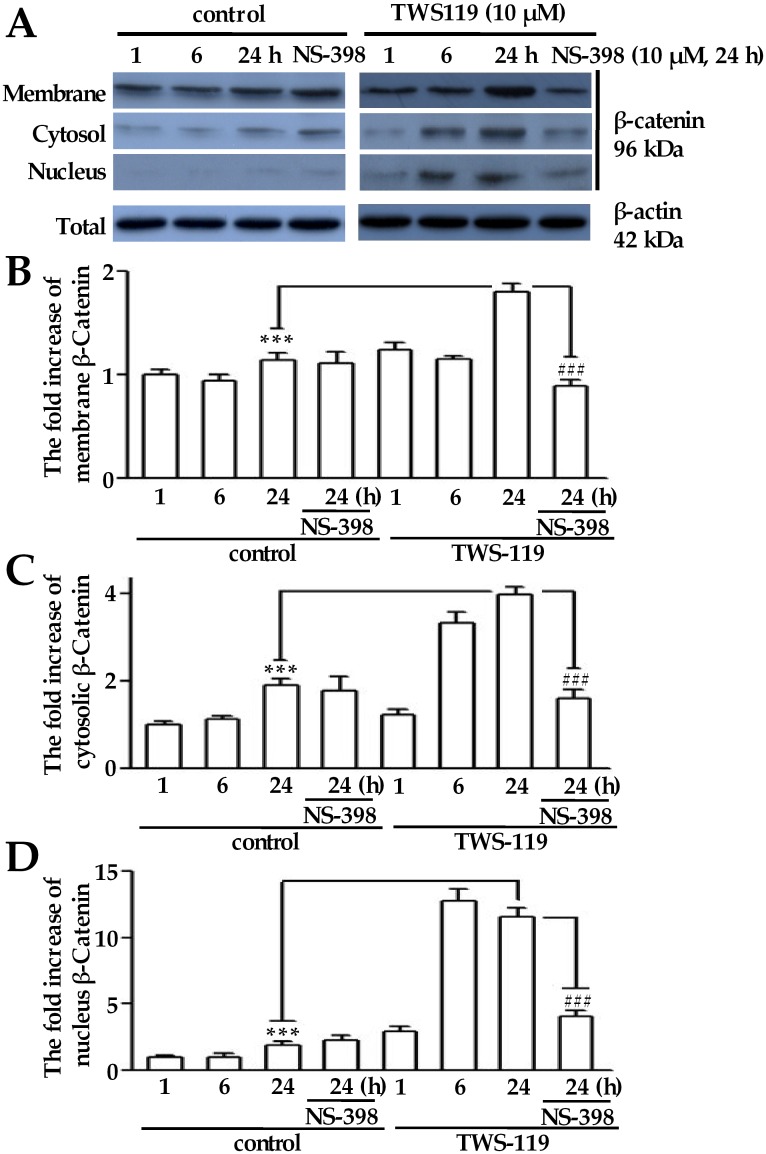
β-Catenin expression induced by TWS119 in the membrane, cytosolic and nucleic fractions from cultured adult rat DRG cells. (A) Representative blots of the β-catenin expression in the membrane, cytosolic and nucleic fractions from cultured DRG cells exposed to TWS119 with or without NS-398 (1 μM) for 1 to 24 h. The fold increases of the β-catenin expression have been quantified by normalizing the data to its expression level of the membrane fraction (B), the cytosolic fraction (C) and the nucleic fraction (D) in the control at 1 h. *** denotes *p* < 0.001 compared to the β-catenin expression change of the control in the membrane, cytosol and nucleus at 24 h; ### denotes *p* <0.001 compared to the β-catenin expression change of the NS-398 in the membrane, cytosol and nucleus at 24 h (n = 5; one-way repeated-measures analysis of variance, followed by Newman-Keuls post hoc test).

To clarify the relationship between β-catenin and COX-2, we further mimicked the Wnt/β-catenin signaling in cultured DRG cells with either TWS119 (a distinct GSK-3β inhibitor) or Wnt-3a (an agonist of β-catenin transcriptional activity), in combination with or without NS-398 (a selective inhibitor of COX-2) or Dkk1 (an antagonist of the Wnt/β-catenin signaling) [18,19]. As shown in Fig 2, the total protein level of β-catenin increased in a time-dependent manner after TWS119 treatment. The fold increases of β-catenin expression in the membrane, cytosolic and nucleic fractions increased 1.80 ± 0.08, 3.98 ± 0.19, and 11.58 ± 0.65 in TWS119 treated DRG cells, respectively, compared to those in the corresponding fractions of control cells (1.14 ± 0.07, 1.90 ± 0.16, and 1.92 ± 0.29, respectively) at 24 h. The NS-398 treatment reduced the TWS119-induced β-catenin expression in the membrane, cytosolic and nucleic fractions to a certain degree (0.89 ± 0.07, 1.61 ± 0.18 and 4.08 ± 0.43, respectively) at 24 h (Fig 2B, 2C and 2D).

**Fig 3:**
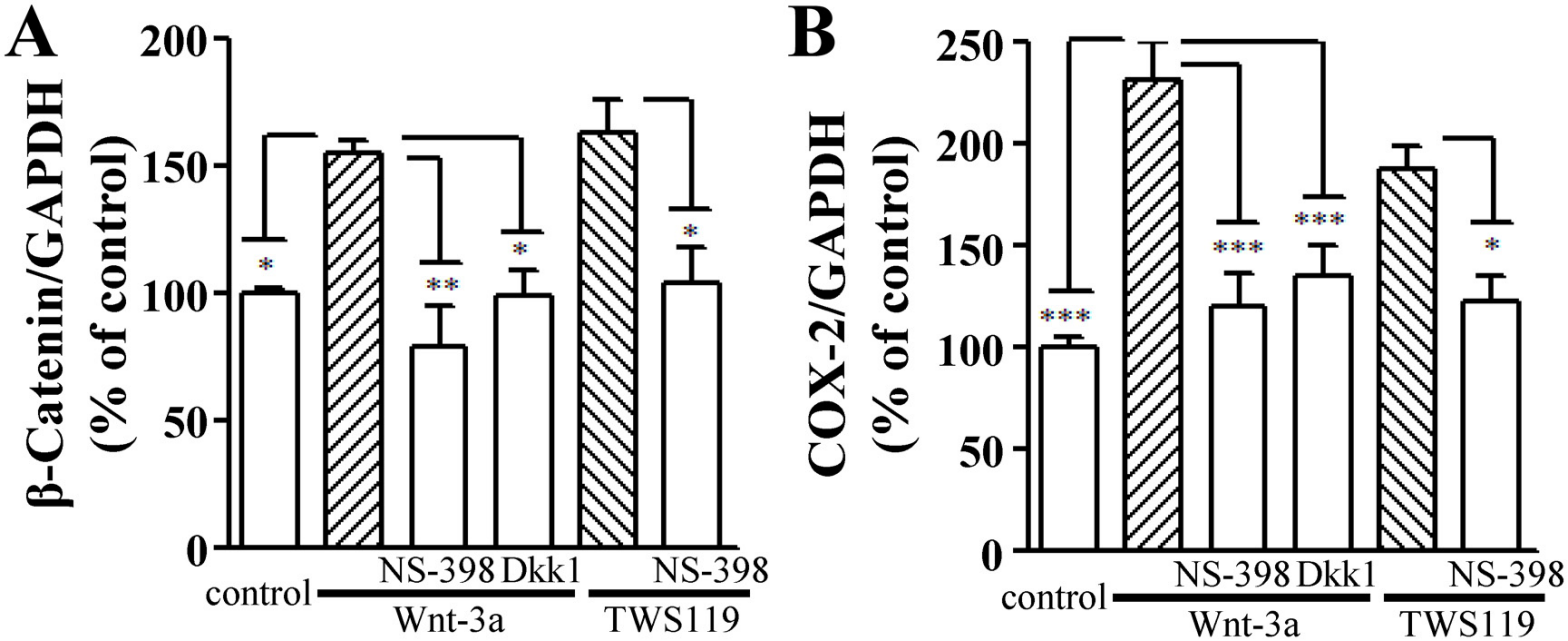
Effects of TWS119 or Wnt-3a on the gene transcription level of *β-catenin* and *COX-2* in cultured DRG cells. The influences of TWS119 (10 μM) or Wnt-3a (10 ng/ml; 24 h) with or without Dkk1 (100 ng/ml) or NS-398 (1 μM) on the mRNA levels of *β-catenin* (A) and *COX-2* (B) in cultured DRG cells (one-way analysis of variance, followed by Newman-Keuls post hoc test). *, ** and *** denote *P* < 0.05, 0.01 and 0.001, respectively (n = 5).

Interestingly, both Wnt-3a and TWS119 also increased the mRNA expressions of *β-catenin* (155 ± 5% and 163 ± 13% of the control, respectively) and *COX-2* (231 ± 19% and 188 ± 12% of the control, respectively) in cultured DRG cells in a Dkk1-sensitive manner (99 ± 10% and 136 ± 15% of the control, respectively; Fig 3A and 3B). Furthermore, the increased mRNA levels of *β-catenin* and *COX-2* evoked by either TWS119 or Wnt-3a were significantly inhibited by pre-treatment of NS-398 (Fig 3). The results shown in Figs 2 and 3 indicate that both β-catenin and COX-2 are downstream of Wnt/β-catenin signaling pathway and reciprocally regulated in DRG cells.

### Nuclear accumulation of β-catenin stimulates the biosynthesis and release of substance P

**Fig 4:**
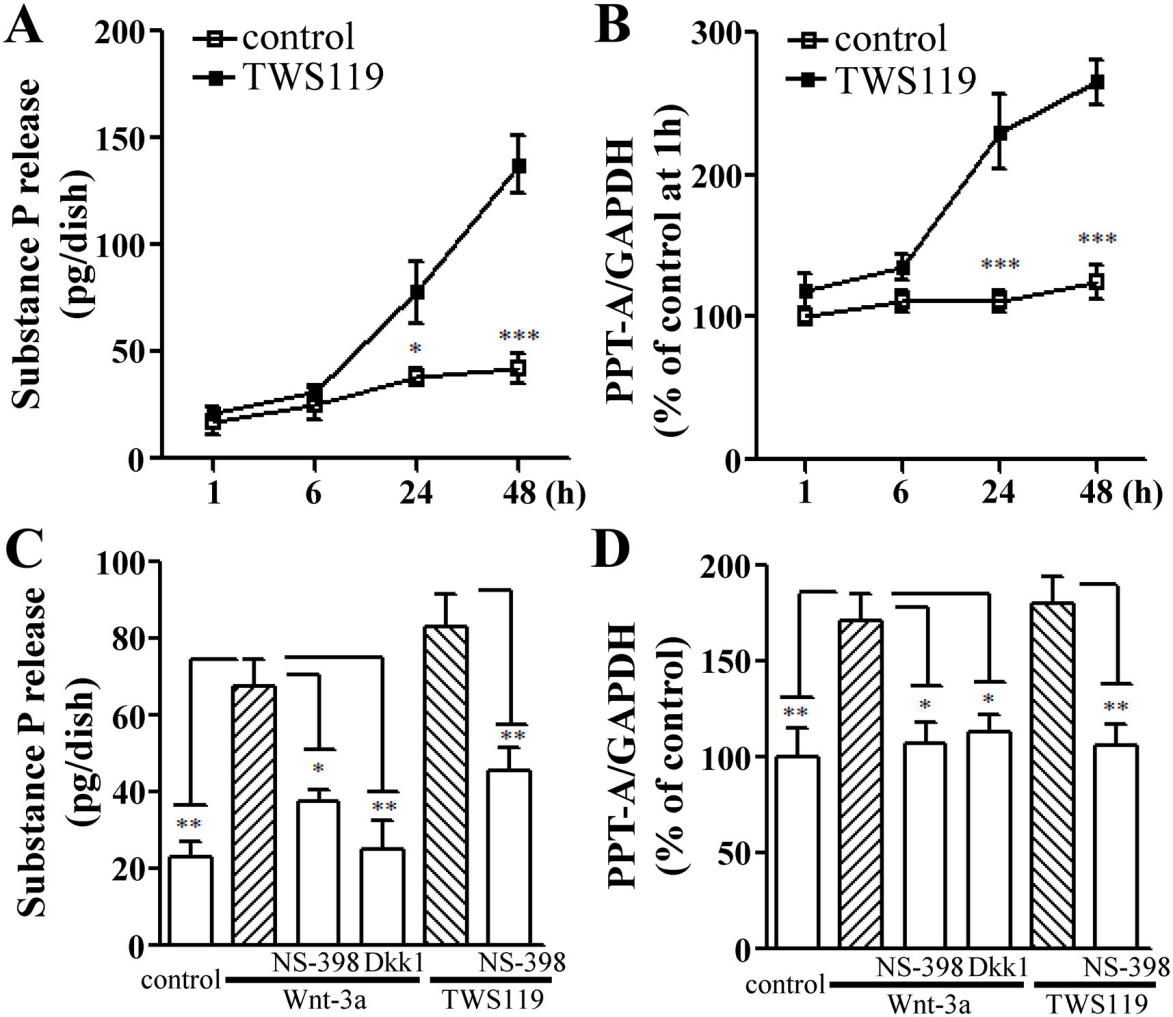
Effects of TWS119 or Wnt-3a on the biosynthesis and release of substance P in cultured DRG cells. The time-course of the release of substance P (A) and *PPT-A* mRNA expression (B) induced by TWS119 (10 μM) from cultured DRG cells (two-way repeated-measures analysis of variance, followed by Bonferroni post hoc test). The influences of TWS119 (10 μM) or Wnt-3a (10 ng/ml; 24 h) with or without Dkk1 (100 ng/ml) or NS-398 (1 μM) on the substance P release (C) and the mRNA levels of *PPT-A* (D) in cultured DRG cells (one-way analysis of variance, followed by Newman-Keuls post hoc test). *, ** and *** denote *P* < 0.05, 0.01 and 0.001, respectively (n = 5).

Substance P is a specific indicator of C-fibber neurons, and plays an important role in pain signal transmission by potentiating nociceptive signaling and revealing pain-related behavior [16,20]. Our previous data indicated that the synthesis and release of substance P can be blocked by inhibition of COX-2 induction in the L5 DRG of CCI neuropathic rats. Hence, we tended to focus our attention on whether β-catenin is involved in the biosynthesis and release of substance P in cultured DRG cells. As expected, the TWS119 treatment also increased the substance P release and its gene transcription level (*PPT-A* mRNA) in a time-dependent manner within 48 h (Fig 4A and 4B). To further confirm the effect of β-catenin nuclear accumulation on the biosynthesis and release of substance P in cultured DRG cells, we used a Wnt/β-catenin signaling agonist Wnt-3a [21]. As shown in Fig 4C, the increased release levels of substance P by both Wnt-3a (67 ± 7 pg/dish) and TWS119 (83 ± 9 pg/dish) were significantly attenuated by pre-treatment with either Dkk1 (38 ± 3 pg/dish) or NS-398 (25 ± 8 and 46 ± 6 pg/dish, respectively).

Consistent with the findings of substance P release, exposure of DRG cells to either Wnt-3a or TWS119 obviously enhanced the gene transcription level of substance P (Fig 4B and 4D). The increased mRNA level of *PPT-A* by both Wnt-3a (171 ± 13% of the control) and TWS119 (180 ± 14% of the control) were significantly attenuated by pre-treatment with either Dkk1 (113 ± 9% of the control) or NS-398 (108 ± 10% and 106 ± 11% of the control, respectively; Fig 4D). These data suggest that the activation of Wnt/β-catenin signaling is involved in the biosynthesis and release of substance P in a COX-2-dependent manner.

## Discussion

In the present study, we demonstrated that the activation of β-catenin signaling by either CCI operation or its activators (TWS119 or Wnt-3a) modulates the biosynthesis and release of substance P release from DRG cells through an induction of COX-2.

As an inducible enzyme pivotal in the inflammatory response, COX-2 converts arachidonic acid to the prostaglandins required for the biosynthesis and release of substance P during the inflammatory process [16,22,23]. Moreover, prostaglandin E_2_ has been reported to induce the accumulation of nuclear β-catenin in human colon cancer cells [14]. Purified Wnt-3a significantly enhanced *COX-2* mRNA expression in a dose- and time-dependent manner [24,25]. In addition, as a target of Wnt/β-catenin signaling pathway [26], HMGA1 was proved to directly bind to the promoter of COX-2 and activate its transcription in human umbilical vein endothelial cells [27] and pancreatic epithelial cells [28,29], suggesting the existence of an indirect activation of COX-2 expression by Wnt/β-catenin signaling pathway. Interestingly, the present results confirmed aberrant nuclear accumulation of β-catenin was accompanied with up-regulation of both COX-2 protein level and the biosynthesis and release of substance P in ipsilateral L5 DRGs of CCI rats, as our previous paper with the same paraffin-embedded L5 DRG sections of CCI rats [16]. Compared to the above observations, our present findings strongly suggest that the enhancement of pain behaviors after nerve injury requires the induction of COX-2 and the nuclear accumulation of β-catenin.

Moreover, the COX-2 inhibition abolished the *β-catenin* mRNA expression and/or its protein translocation induced by the CCI operation or Wnt/β-catenin signaling activators (Figs 1 and 2), indicating the existence of a positive feedback loop between COX-2 and β-catenin for the biosynthesis and release of substance P release in our present study. This appearance of a loop between COX-2 and β-catenin could be explained by the COX-2 promoter containing a functional TCF/LEF-response element for the enhancement of Wnt/β-catenin signaling [24].

To address the relationship among the activation of COX-2, the expression of β-catenin and the biosynthesis and release of substance P, we therefore mimicked the activation of β-catenin signaling pathway to induce the nuclear translocation of β-catenin in cultured rat DRG cells according to a previously described method using a GSK-3β inhibitor TWS119 and a Wnt signaling agonist Wnt-3a. Notably, our *in vitro* experiments indicated that the β-catenin activation by both TWS119 and Wnt-3a resulted in the up-regulation of *PPT-A* mRNA expression and the substance P release from cultured rat DRG cells. As expected, the increases in the biosynthesis and release of substance P evoked by either TWS119 or Wnt-3a could also be inhibited by a selective COX-2 inhibitor NS-398 (Figs 2 and 4). In addition, the long-term exposure of DRG cells to both TWS119 and Wnt-3a resulted in similar response patterns for *COX-2* and *β-catenin* mRNA levels in comparison with those in the control (Fig 3). Namely, in agreement with the effect of Wnt-3a, inhibition of GSK-3β by TWS119 may initiate the inflammatory responses through the induction of COX-2 in primary afferent nerve. Furthermore, an antagonist of the Wnt/β-catenin signaling Dkk1 significantly attenuated the increases in the *PPT-A* mRNA level and the release of substance P in cultured DRG cells treated with Wnt-3a (Figs 3, 4C and 4D) suggesting the direct regulatory role of β-catenin signaling on *PPT-A* gene transcription and substance P release. Of course, our previous studies demonstrated that the release of substance P may be also triggered by itself through the activation of its receptor and the de novo protein synthesis of COX-2 in cultured DRG cells [3]. In addition, recent reports indicated that substance P enhanced the proliferation of bone marrow stromal stem cells of rats via regulation of β-catenin signaling [30].

There were some interesting discrepancies or observations in our study: (1) As shown in Table 1, in ipsilateral DRGs after CCI treatment, β-catenin located more in the cytosol and nucleus. Meanwhile, more β-catenin were observed on the membrane in contralateral DRGs. This reversed pattern of β-catenin localization was unexpected. We speculate that CCI surgery could enhance the expression level of β-catenin in contralateral DRGs due to some unknown mechanism as shown in Fig 1B. Therefore, as the membrane pool of β-catenin is limited, those over-expressed β-catenin will be located in cytosol and nucleus, and hence cause a decrease in the percentage of membrane-localized pattern in contralateral DRGs. (2) Diclofenac was capable of inhibiting CCI induced increase of β-catenin expression in both ipsilateral and contralateral L5 DRGs, as shown in Fig 1B. It has been suggested that diclofenac is capable of inhibiting COX-2 activity [31]. Therefore, this observation further enhanced the possibility that there is a close relationship between COX-2 and β-catenin. (3) We observed increases of the β-catenin in all fractions in Fig 2B, instead of an increase in nucleic fraction along, which is consistent with previous research [32]. This phenomenon is partly, if not all, due to the activation mechanism of β-catenin. Briefly, under inactivated state, most β-catenin are mobilized on the membrane and the free monomers are phosphorylated by casein kinase Iα (CKIα) and glycogen synthase kinase 3 (GSK3), and subsequently degraded after ubiquitination. However, upon activation, CKIα and GSK3 are inactivated and hence more and more β-catenin will accumulate in cytosol and nucleus. As a direct consequence, expression of β-catenin in membrane, cytosol and nucleus fraction will be increased simultaneously.

**Fig 5:**
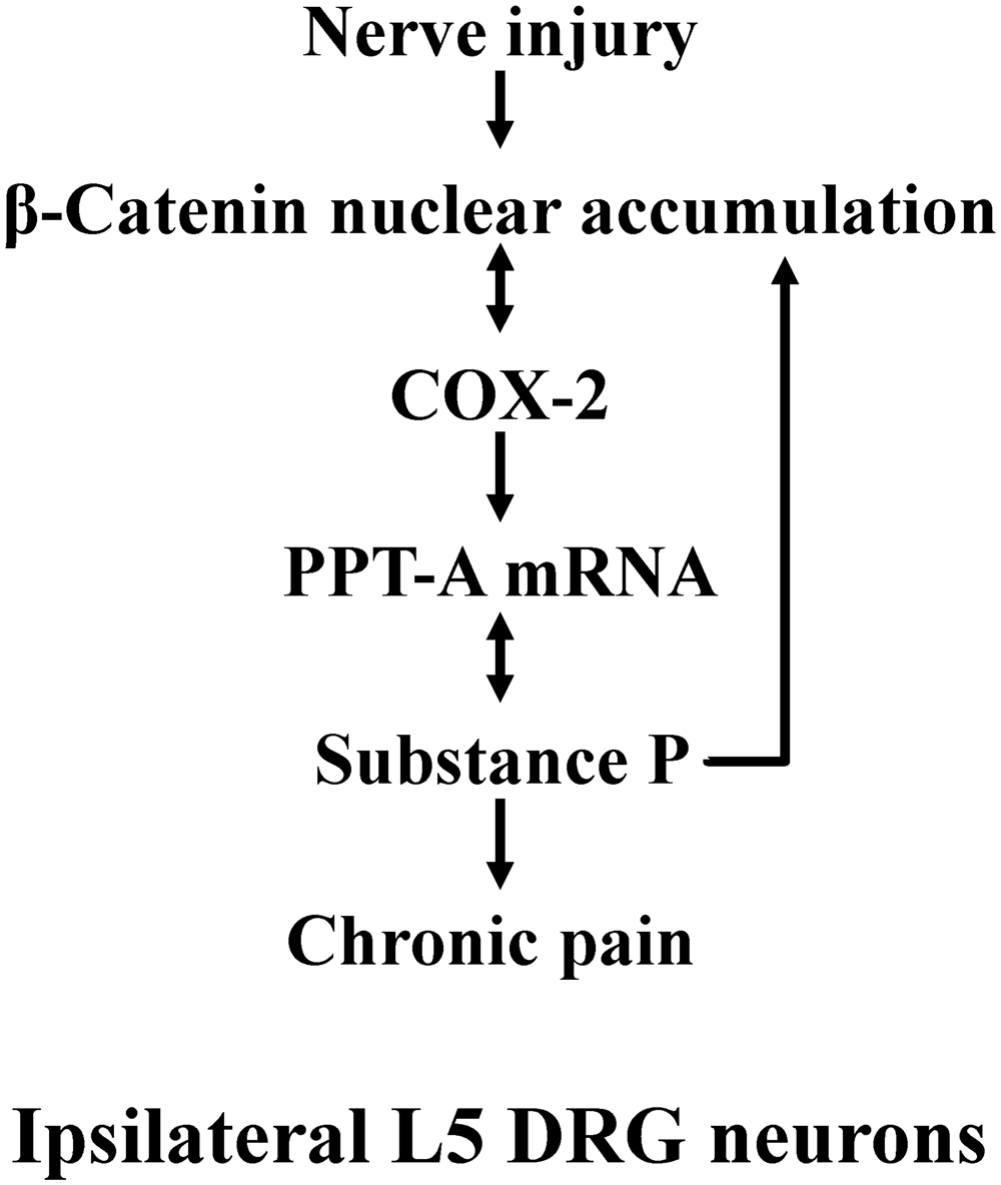
Schematic diagram showing the possible mechanisms by which nerve injury activates β-catenin signaling to induce the release and synthesis of substance P in ipsilateral L5 DRG.

Based on our present results from *in vivo* and *in vitro* experiments and the above-mentioned observations reported previously, we herein considered that the possible molecular mechanisms of chronic pain by which the β-catenin signaling was required for evoking the biosynthesis and release of substance P (Fig 5). Instead, the aberrant activation of β-catenin signaling evoked by peripheral nerve injury may promote the induction of COX-2. Adversely, the induced COX-2 also stimulates the β-catenin signaling again, and then the crosstalk between β-catenin and COX-2 may contribute to the maintenance of nociceptive information. As a result, aberrant synthesis and release of substance P are evoked from DRG neurons. Following the reduction of substance P level in intracellular substance P pools evoked by the released substance P, a signal will be elicited to convey information for the transcripts of nuclear *PPT-A* genes to increase the substance P synthesis [20]. The released substance P may bind to its receptor (neurokinin-1 receptor), eliciting a signal to further release the substance P [3] and enhancing further up-regulation of β-catenin signaling [30]. Just the release and synthesis of substance P are abnormally evoked by the crosstalk between β-catenin and COX-2 as tonic peripheral nociceptive input contribute tremendously to the initiation and maintenance of central sensitization states. However, the detailed role of β-catenin in initiating and maintaining chronic pain responses in primary afferent neurons will be addressed in the future by facilitating the over-expression and/or knock-down techniques.

## Conclusion

The current studies demonstrated that a novel crosstalk between β-catenin and COX-2 plays an important role in regulating the biosynthesis and release of substance P, which may contribute to the inflammation responses related to chronic pain.
